# Blind-sided by privacy? Digital contact tracing, the Apple/Google API and big tech’s newfound role as global health policy makers

**DOI:** 10.1007/s10676-020-09547-x

**Published:** 2020-07-18

**Authors:** Tamar Sharon

**Affiliations:** Faculty of Philosophy, Theology and Religious Studies, Radboud University, PO Box 9103, 6500 HD Nijmegen, The Netherlands

**Keywords:** Digital contact tracing, Big tech, Privacy, Justice, Digital ethics, COVID-19

## Abstract

Since the outbreak of COVID-19, governments have turned their attention to digital contact tracing. In many countries, public debate has focused on the risks this technology poses to privacy, with advocates and experts sounding alarm bells about surveillance and mission creep reminiscent of the post 9/11 era. Yet, when Apple and Google launched their contact tracing API in April 2020, some of the world’s leading privacy experts applauded this initiative for its privacy-preserving technical specifications. In an interesting twist, the tech giants came to be portrayed as greater champions of privacy than some democratic governments. This article proposes to view the Apple/Google API in terms of a broader phenomenon whereby tech corporations are encroaching into ever new spheres of social life. From this perspective, the (legitimate) advantage these actors have accrued in the sphere of the production of digital goods provides them with (illegitimate) access to the spheres of health and medicine, and more worrisome, to the sphere of politics. These sphere transgressions raise numerous risks that are not captured by the focus on privacy harms. Namely, a crowding out of essential spherical expertise, new dependencies on corporate actors for the delivery of essential, public goods, the shaping of (global) public policy by non-representative, private actors and ultimately, the accumulation of decision-making power across multiple spheres. While privacy is certainly an important value, its centrality in the debate on digital contact tracing may blind us to these broader societal harms and unwittingly pave the way for ever more sphere transgressions.

## Introduction: contact tracing and the automation of a public health practice

Since the outbreak of the COVID-19 pandemic in early 2020, governments and health authorities around the world have attempted to mobilize digital technologies to address this novel threat, including the use of tracker wristbands, smartphone applications, thermal cameras, facial recognition and drones (The Economist 2020). In the prolonged anticipation of more permanent solutions like a vaccine, contact tracing apps in particular are being explored as tools to help contain the spread of the virus (EC 2020; WHO 2020). Contact tracing is a time-tested method that has been successfully used to fight infectious disease outbreaks including syphilis, measles, HIV and Ebola. It involves identifying infected individuals and informing the people they have been in contact with that they are at risk, through a meticulous process of retracing where and with whom an infected individual has been in proximity. Automated contact tracing offers several advantages over traditional contact tracing in the case of the COVID-19 pandemic (CDC 2020a; Ferreti et al. 2020). First, it seeks to automate a labor-intensive practice in a situation where there is a scarcity of human contact tracers. Moreover, it may offer more accuracy where human memories are fallible—particularly in the case of COVID-19, where infection can be asymptomatic for up to two weeks. The speed of contagion of the COVID-19 virus, finally, requires swift contact tracing in order to be effective. Digital contact tracing seeks to address these limitations, by providing speed, scale and accuracy.

As with many attempts at automation, numerous obstacles impede the path to smooth, seamless digital contact tracing. It is not at all clear, in the first instance, that these contact tracing apps will be effective (Ada Lovelace Institute 2020). In countries where digital contact tracing was first deployed, such as China, Singapore and South Korea, the actual role of this technique in controlling the spread of infections is ambiguous (Frieden 2020). Accuracy is another major concern here. Bluetooth, currently the preferred technology for digital contact tracing, can result in high amounts of false positives, by picking up “contacts” that are not epidemiologically significant (Lee 2020; Vaughn 2020). Effective digital contact tracing also relies on a high level of uptake by the population, which will be difficult to ensure if these systems are to be voluntary (Hinch et al. 2020). This issue is complicated by the question of *who* can participate in digital contact tracing. Not everyone has access to a smartphone, even in wealthier nations. And of those who do, an estimated 1.5 billion people globally still use basic phones that do not have the necessary technical requirements, such as Bluetooth “low energy” chips, that are being used in many contact tracing apps (Counterpoint 2020). Importantly, these populations tend to be lower socio-economic groups and older people, exactly those people who are also among the most vulnerable to the virus (O’Neil 2020). While these limitations have dampened some of the initial enthusiasm around digital contact tracing as an easy solution to curbing the spread of the virus, the technology is still seen as an important complement in national post-lockdown strategies. At the time of writing, at least 80 contact tracing systems have been launched or are in development around the world,^1^ and supra-national bodies like the European Commission and the WHO are publishing guidelines for app development, or developing their own (EC 2020; Dave 2020).

Beyond these more practical questions, one of the major points of contention in the implementation of digital contact tracing has been its potential to cause harm through privacy breaches (Ienca and Vayena 2020; McGee et al. 2020). In Europe and the United States, in particular, where public awareness on the use of digital surveillance for public interests has gained a heightened sensitivity to privacy issues since the Snowden revelations, this triggered a vigorous public debate on the need to develop privacy-friendly digital contact tracing. Yet, when Apple and Google—corporations whose data practices are typically the focus of ethical debate—launched their contact tracing API in April 2020, some of the world’s leading privacy experts applauded this initiative for its privacy-preserving technical specifications. In an interesting twist, the tech giants came to be portrayed as greater champions of privacy than some European governments.

This article explores what else is at stake when two of the world’s most powerful corporations move into the field of pandemic response management, even when this is done in a privacy-preserving manner. Drawing on Michael Walzer’s (1983) theory of justice, and the autonomy of spheres of social life as a precondition for equality and justice, I propose to view the Apple/Google API in terms of a broader phenomenon of powerful tech corporations encroaching into ever new spheres, by virtue of the fact that their digital expertise has become a coveted currency in almost all spheres of life. From this perspective, the (legitimate) advantage that tech companies have accrued in the sphere of the production of digital goods provides them with (illegitimate) access to the spheres of health and medicine, and more worrisome, to the sphere of politics. Each of these transgressions, I explain, poses specific risks that are not captured by the focus on privacy harms. Encroachment into the sphere of health and medicine can lead to a crowding out of significant traditional sectorial expertise and the reorganization of health and medicine in line with the values and interests of corporate actors. Encroachment into the sphere of politics can lead to new dependencies on corporate actors for the delivery of essential public goods, often underpinned by relationships of charity and gratitude rather than justice and duty, and ultimately to the shaping of public policy by non-elected, unaccountable actors. The overall risk is an accrual of advantage and power across spheres—what Walzer calls tyranny. While privacy is an important intrinsic and instrumental value, the centrality that it has received in the debate on digital contact tracing, and arguably in other debates on digitalization, may blind us to these broader societal harms and unwittingly pave the way for ever more sphere transgressions.

## Lessons learned: privacy takes central stage

### Mission creep

Beyond the pressing question of whether digital contact tracing will actually prove to be effective, public debate in many countries has focused on the risks contact tracing apps pose to privacy.^2^ While it is clear that, in times of crisis, governments may need temporary powers that suspend some civil liberties and that the sharing of sensitive data like one’s health status and location can contribute to containing the spread of the virus, the use of digital methods for doing so introduces novel risks. Namely, the ease by which digital data, as opposed to data in the paper age, flow between contexts that are governed by different norms of privacy (Nissenbaum 2010), has given rise to new fears of “mission creep”. For example, data on people’s health status collected for contact tracing could be used for other purposes and by third parties: for determining who can and cannot get back to work, or for determining who can and cannot access public spaces like subways, malls and markets (Morley et al. 2020; Parker and Jones 2020).^3^ Location data, similarly, can be used to show who a person associates with and to infer what they were doing at a given time, fear of which can have a chilling effect on people’s participation in certain activities (Rahman 2020). Privacy here needs to be addressed from both the angle of the person who is infected and the person who is at risk of infection because they have been in contact with the infected person.

Within the public discussions around digital contact tracing, these concerns have tended to be framed in terms of a trade-off between individual privacy rights and public health. The European General Data Protection Regulation supports this framing with specific articles that provide the legal grounds for processing personal data in the context of epidemics. Article 9, for example, allows the processing of personal data “for reasons of public interest in the area of public health, such as protecting against serious cross-border threats to health”, provided that such processing is proportionate to the aim pursued, respects the right to data protection and safeguards the rights and freedoms of the data subject. But many concerns have been raised about just how much privacy should be traded off for public health, and if this dichotomous framing is actually an accurate depiction of the considerations at hand (Goldenfein et al. 2020). Importantly, as privacy advocates and civil liberties campaigners have pointed out (Ross 2020; Schwartz 2020), the “privacy vs. public health” framing is reminiscent of the counterterrorism debate that followed the 2001 attacks on the World Trade Center, which was framed in terms of “privacy vs. security”. In that debate, the tangible threat of terrorism justified an expansion of governments’ surveillance powers (whose effectiveness was also questionable), and the creation of a global surveillance behemoth that persists to this day. Similarly, scholars question what will happen to the epidemiological surveillance constellation that contact tracing apps support once the pandemic is over, and if the erosion of privacy will become part of a permanent state of vigilance against new viruses (McGee et al. 2020; Roth et al. 2020). Prompted by these concerns, a number of advocacy and advisory groups and governing bodies have indicated the need to develop contact tracing apps in a way that would be privacy preserving, with an emphasis on voluntariness, transparency, collection and sharing of non-traceable identifiers and de-centralized storage (see e.g. EC 2020; EDPS 2020; Ada Lovelace Institute 2020; NCB 2020).

### Contact tracing privacy by design

For these critical experts, the trade-off between privacy and public health is a false one. Furthermore, it is possible to translate the value of privacy, as well as other ethical and legal principles, into technological design (Hildebrandt and Tielemans 2013), thus imposing material restrictions on possible excesses in times of crisis, and all the while promoting public health. Design choices have therefore been foregrounded in the discussion on digital contact tracing.

First and foremost, the use of low-energy Bluetooth has been advanced as an important design solution. A number of the early digital contact tracing systems, for example the ones used in China and South Korea, but also in India, Israel and Iceland, use location data from phones. Automated GPS tracking does not only lack in precision, it is typically non-consensual and scores low on privacy. Bluetooth-based apps, conversely, avoid tracking the location of users and are perceived as less intrusive. Here, phones generate random numerical IDs or “handshakes” that are transmitted to nearby devices, which commit these to a contact history log. If a person experiences symptoms or tests positive, this will send a notification to the devices whose identifiers it had previously received. Because these handshakes are encrypted, and because users’ location is not logged, Bluetooth has been portrayed as superior to location tracking for privacy reasons, and has been recommended by the EC guidance for app development (EC 2020) and favored by many European governments.

Another important criterion for many privacy experts has been that contact tracing apps rely on systems that are decentralized. Centralized apps, used for example by Singapore and Australia, send data collected by a user’s phone to a central database controlled by a national health agency or other governmental authority. This authority then works out who to send an alert to among the contacts that an infected person’s phone has registered. This concentration of data and power has been a crucial point of contention for privacy advocates, who have widely supported decentralized systems. In this model the data collected by phones are not sent to a central server but are stored locally, on individual phones, and the phones do the contact-matching themselves—no central authority needs to be involved. The “Decentralized Privacy-Preserving Proximity Tracing”, or DP-3T, protocol was an important frontrunner in this regard (Troncoso et al. 2020), developed by a group of European academics in response to the early “Pan-European Privacy-Preserving Proximity Tracing” consortium which backed a centralized system. DP-3T triggered a debate on centralized vs. decentralized systems which became highly political (Criddle and Kelion 2020),^4^ and resulted in many countries, such as Germany, Austria, Estonia and Switzerland, opting for decentralized models, and the European Parliament (2020) and the CDC (2020b) calling for apps to be decentralized.

### Apple and Google: the unexpected champions of privacy-friendly contact tracing

Amidst these heated debates, in early April 2020 Apple and Google revealed that they, too, were busy developing contact tracing technology (Apple 2020). In a first instance the Apple/Google software consists of an API that allows Apple and Android phones to exchange data with each other. Users have to download contact tracing apps that use the API as the underlying system. At a later stage, contact tracing software will be added directly into the operating systems of phones as a default. To many, the Apple/Google initiative came as a surprise. Not just because the plans had remained covert, or because the two companies habitually tend to be competitors more than collaborators, but because their initiative put privacy center stage. The questionable data practices that these technology corporations are known for—Google admittedly much more than Apple—would not normally inspire trust for such a sensitive technology like digital contact tracing. Likewise, one of the big lessons learned from post-9/11 surveillance creep was the importance of the merging of military and corporate interests driving the political economy of surveillance (Ball & Snider 2013). And yet, the Apple/Google API has been presented as a response to some of the gravest privacy concerns voiced in the digital contact tracing debate. Indeed, the draft technical documentation that the companies quickly released showed that many of the specifications of the proposal incorporated requirements spelled out by privacy experts for secure contact tracing, and had even been inspired by the ultra privacy-sensitive DP-3T protocol (Leprince-Ringuet 2020). These include: the use of Bluetooth (no need for location data); the generation of random identification numbers by phones that change every 10–20 min (no personally identifiable data is exchanged); an opt-in system (users have to consent to their device broadcasting their identifiers once they’ve tested positive); and the cherry on top—a decentralized model (data is stored and processed on users’ devices).

This alignment with privacy specifications led to widespread endorsement and applause for the Apple/Google initiative by some of the leading privacy experts in the contact tracing debate. For example, the European Data Protection Supervisor immediately backed the initiative, stating in a tweet that “it seems to tick the right boxes as regards #user choice, #data protection by design and pan-European #interoperability”.^5^ And researchers behind the DP-3T protocol praised the compatibility of the Apple/Google API with their own. As Marcel Salathé, one of the researchers who helped write DP-3T commented, “For us (…) it was a no-brainer. Most of the things we had proposed with DP-3T were in Apple and Google’s API” (in Leprince-Ringuet 2020). The Apple/Google API’s privacy preserving specifications, along with the promise for greater interoperability across countries—something that the European Commission promoted as crucial since initial discussions on digital contact tracing—were persuasive, and more and more governments announced that they would adopt it. These include [at the time of writing] Austria, Canada, Denmark, Estonia, Germany, Ireland, Italy, Japan, Latvia, Malaysia, Northern Ireland, and Switzerland.^6^ In an interesting twist, the tech giants came to be portrayed as greater champions of privacy than some democratic governments, and as what one privacy consultant called “the most efficient privacy regulators in the world” (in Scott et al. 2020).

Not all privacy experts agree about the level of privacy protection the Apple/Google API will deliver. For Jaap-Henk Hoepman (2020) for example, embedding the contact tracing functionality in the operating system layer creates a dormant functionality for mass surveillance, whereby the contact tracing microdata are under the control of Apple/Google. Furthermore, Hoepman explains, the platform can easily be transformed into a centralized form of tracing, and may allow malicious apps to learn which people an infected person has been in contact with. Others are calling for the implementation of additional safeguards, such as careful auditing and mechanisms for ensuring the technology can be uninstalled once the pandemic is over. Others still are more generally suspicious of the Bluetooth technology the API makes use of, which operates in the background without users noticing or knowing what happens exactly to their data.^7^ But this focus on privacy, while certainly important, risks blindsiding us to the bigger questions at hand. Even if the Apple/Google contact tracing technology does get the privacy issue just right, what other trade-offs are involved in letting these companies contribute to the development and deployment of what might be the largest scale crisis management measure for the pandemic so far?

## The Googlization of pandemic response

To answer this, it is helpful first to note that the development of digital contact tracing is only one of many ways that the large tech corporations, not just Apple and Google, but also Facebook, Amazon, Microsoft, other subsidiaries of Alphabet, as well as their Asian counterparts including Alibaba, Baidu Tencent and Huwaei, have contributed to addressing the threats posed by COVID-19. In addition to providing key information and resources on COVID-19 on their respective platforms, many of these companies began developing COVID specific data collection, data sharing and data analysis tools, or have earmarked significant amounts of funds for COVID related research, early on during the pandemic.

### Tech companies and the COVID-19 threat

In March 2020 already, Facebook added COVID specific “Disease Prevention Maps” to its “Data for Good” program, for sharing user location data with researchers seeking to identify virus hotspots (Facebook 2020). Also early on, Verily, Alphabet’s life sciences subsidiary, launched a screening and testing website where users could fill out a multi-question survey about their symptoms and get directed to a drive-through testing location, run by Alphabet, Verily and Google volunteers. Originally limited to the state of California, the number of testing sites soon expanded across the US, to include 130 sites in 12 states at the time of writing. Apple contributed to a similar triage effort in California by building an app with Stanford Medicine to connect firefighters, police officers and paramedics to testing sites (Leswing 2020). In the UK, Amazon, Microsoft, Google and Palantir are assisting the National Health Service (NHS) in setting up a “COVID-19 data store”, to track how hospitals are managing beds, capacity oxygen and ventilators, and to help them allocate resources appropriately (Fitzgerald and Crider 2020).

Almost all of the large tech companies, furthermore, are contributing in some way or other to research and clinical efforts on COVID-19. IBM, Amazon, Google and Microsoft have contributed computing power, necessary to process very large datasets in epidemiological, bioinformatics and molecular modelling. Google Cloud, for example, has mobilized $20 million in Google Cloud credits to support academic research (Kurian 2020). Baidu and Google’s DeepMind are applying deep-learning techniques for modeling the structure of the coronavirus’ proteins, which could be useful in developing a vaccine (DeepMind 2020). And Microsoft and Facebook, via the Chan Zuckerberg Initiative, have helped compile datasets of COVID-19 related research papers for easy query. An interesting case in point is Verily’s coronavirus screening website. Initially set up to help triage individuals at high risk in a context of limited risk screening and testing capacity, the initiative soon fused with Verily’s Project Baseline. The Project Baseline is an ambitious project set up some years ago that seeks to create a “baseline” of human health by aggregating a wealth of clinical, genomic and lifestyle data donated by healthy volunteers. The dataset will be used for research purposes and the platform as a means of connecting potential participants with clinical research. Shortly after Verily launched its screening tool, it began calling the initiative the “Baseline COVID-19 Pilot Program”. What this indicates is that the triage tool will soon be used for much more than just triage and testing, most likely for channeling users towards enrollment in trials, such as the serology study that Verily announced as the first of its Baseline COVID-19 Research Project initiatives (Verily 2020). Verily could thus quickly leverage an already existing infrastructure for data collection for biomedical research to expand its COVID-19 involvement.

Most of these companies have not only been involved in providing the infrastructure for research to take place, but have also been forthcoming in funding research. The Chan Zuckerberg Initiative has donated over $13 million to a collaboration between UC San Francisco, Stanford University and the Chan Zuckerberg Biohub, to study COVID-19. And the Bill and Melinda Gates Foundation, in particular, has been portrayed in the media as one of the “leaders in the coronavirus response” (Piper 2020). The foundation, with one of the largest endowments of any charity in the world and 20 years of experience in public health and infectious disease response, launched numerous pandemic response endeavors as early as February 2020 and announced in April that it would shift most of its attention to fighting the pandemic (Grothaus 2020). These have included commitments of some $300 million, for home testing kits, a “Therapeutics Accelerator” to study the most effective treatments for the virus, vaccine development (and enhancement of global manufacturing and delivery capacity), and a relentless media tour by Gates himself on the importance of social distancing.^8^ These philanthropic efforts extend to other types of relief responses as well. Amongst others, Google has donated Chromebooks and WiFi hotspots to households in California, to help students with remote schooling during the pandemic, and committed to donating over $800 million to support small and medium sized businesses, health organizations and governments. Apple donated $10 million to a COVID-19 fundraiser organized by the WHO. Amazon, in addition to providing $5 million worth of devices to hospitals, schools and families, including Kindles, Fire tablets and Echo for patient monitoring (Amazon 2020), has also donated $100 million to a large charity that operates food banks around the US. And in what some commentators have called “China’s Big Tech donation spree” (Cerulus 2020) Alibaba and Huwaei provided European hospitals hit early on with protective suits and medical masks.

### Precedents in the Googlization of health

If this capacity on the part of tech corporations to contribute not only financial resources, but also the technical, infrastructural and biomedical expertise required to address the current public health crisis seems surprising, it shouldn’t. In the past decade consumer technology companies have swiftly and surely moved into the health and biomedical sector, positioning themselves as important facilitators of data-driven digital health and medicine, in what can be called a “Googlization of health” (Sharon 2016, 2018). Launched in 2014, Apple’s ResearchKit software, for example, now allows medical researchers to carry out clinical studies using the iPhone, and is currently being used by prominent medical institutions like Yale and Stanford. Verily, in addition to its ambitious Project Baseline, is collaborating in research on Parkinson’s disease in Europe and with pharma companies on clinical trial development. Other Alphabet subsidiaries, like DeepMind, are developing AI for medical diagnostics, with some recent successes in cardiovascular disease, eye disease and breast and lung cancer. Amazon has developed a machine learning tool for the processing of unstructured medical texts and its Alexa voice-assistant is now being used by the UK’s NHS to provide NHS health advice to users at home. A number of these companies are also involved in electronic health record management, employee healthcare, health insurance, and the provision of healthcare services, with Verily’s new opioid addiction clinic open since 2019. Neither is health and medicine the only sector into which these companies have begun making permanent inroads pre-corona. During the same period, we have also witnessed their growing involvement in, amongst others, the sectors of transportation, urban planning, education, and space exploration (see for example Vaidhyanathan (2011) and van Dijck et al. (2019)).

## Sphere transgression: from digital goods to health and medicine

### The dangers of sector creep

In his seminal book *Spheres of Justice* (1983), the political philosopher Michael Walzer elaborates a theory of justice and complex equality based on the autonomy of spheres in which different societal goods—education, welfare, wealth, friendship, political power, etc.—are distributed. While inequality is bound to exist amongst individuals within spheres, Walzer maintains that a just society is one where advantage in one sphere cannot be converted into dominance in another. Thus, while one citizen may be chosen over another for political office, leading to some inequality in the sphere of politics, this advantage should not confer that person any advantages in other spheres, such as superior medical care, access to better schools for her children or greater entrepreneurial opportunities. Such conversions and transgressions between spheres, according to Walzer, are a form of tyranny. They lead to both a loss of meaning and significance of those goods which succumb to the distributive logic of the wrong sphere, as well as to the dominance of some members of society by others.

Walzer did not identify an area of technological production in which digital goods are distributed as a sphere per se. But his notion of complex equality based on the separation of distributional spheres is useful for describing what is happening in a phenomenon like the Googlization of health or of pandemic response.^9^ Indeed, what we are witnessing as these companies move into new sectors is that the technical expertise—in terms of data collection, data analytics and infrastructure development—which confers them a clear and legitimate advantage in the sphere of digital goods, is currently being converted into advantages in other spheres, such as the sphere of health and medicine and the sphere of politics. In a context in which ever more sectors of society are undergoing processes of digitalization and datafication, this is to be expected, and it may result in increased technical efficiency and other benefits in some spheres. However, what Walzer would call its “tyrannical” nature requires urgent attention, and presents risks that are hardly limited to privacy harms.

### The crowding out of essential, “spherical” expertise

The recent push in the health and medical sector to move towards more data-driven personalized medicine (Frohlich et al. 2018) has turned digital expertise into a coveted good in the sphere of health, and has made tech companies attractive partners to collaborate with. In other words, it is not based on the merits of their medical (or epidemiological) expertise that these companies’ novel presence in health and medicine is justified.^10^ This lack of traditional “spherical” expertise is problematic. As scholars in the field of science and technology studies have shown, routine human practices and professional tasks, in the medical and other fields, always involve implicit values, norms and skills which are at risk of being omitted or lost when a practice is standardized and automated (Berg 1997; Timmermans and Mauck 2005). In this understanding, automation amounts to the importation of practices that embody values such as efficiency, optimization and speed—values that are and should be decisive in the sphere of technological production—at the expense of traditional sectorial norms and values, which are not always noticeable to outsiders.

Digital contact tracing is a prime instance of the promise of automation, whereby traditional, often repetitive human tasks—be these checking out supermarket products, welfare benefit allocation, driving or medical decision-making—seek to be augmented if not replaced by more efficient, objective and speedy automated systems. But as a practice, digital contact tracing involves implicit values and skills which are integral to its overall aims and cannot be easily translated into automated processes. These include, first and foremost, the capacity to navigate complex human interaction. The public health workers traditionally carrying out contact tracing are trained to undertake epidemiological detective work to establish which contacts matter for disease contagion. This is based on criteria like the environment that was shared with another person, the kind of activity which was being carried out at the time, and for how long. Replacing this type of inquiry with the exchange of signals via Bluetooth is proving problematic (Ada Lovelace 2020). Some phones can detect signals from up to 30 m, without being able to determine if a signal was transmitted from 1 to 29 m away. Bluetooth technology also cannot account for important obstacles to virus transmission, like walls, through which Bluetooth signals can still be transmitted. Moreover, it can hardly control for environmental variables, like ventilation, or direction of the wind. In other words, what constitutes a “contact” for a smartphone does not always have epidemiological value.

Second, the success of traditional contact tracing rests on the ability of the public health worker to build a relationship of trust with the interviewee. Not only so that people feel safe revealing personal details, but also because contact tracing is as much about *identifying* persons at risk of infection as it is about *providing them with targeted information*. Contact tracers need to deliver public health advice, such as the recommendation to go in to quarantine, in a way that people will listen to and act upon this advice. Human skills, including empathy, patience and understanding, which are demonstrated and enacted in the back-and-forth of conversation between people, are required here, and all but missing in an app notification. Moreover, much of the work of human contact tracers has to do with ensuring that people have the material conditions required to sustain a 14-day quarantine, including food in their homes, the ability to care for children who may need to be removed, how to isolate in small spaces and when to seek medical attention (Bourdeaux, Gray and Grosz 2020; Ross 2020). For all of these reasons, for those familiar with the practice, contact tracing has been called “an art as much as a science” (Otterman 2020), which cannot be easily replaced by an app, no matter how accurate or widely used it would be. Here, the importance of keeping a human “in” (or “on top of”) the loop, as critical scholars of algorithms rightly argue for, is not just about avoiding pernicious feedback loops and algorithmic discrimination, but also about carefully acknowledging everything that goes into making a practice “good” (Mol 2008; Pols 2012) before rushing to automate its most self-evident components.

The crowding out of spherical, here epidemiological, expertise has been to an important extent further facilitated by the over-simplified equation of decentralized approaches with privacy-preservation, and centralized approaches with government surveillance; an opposition in part promoted by Apple and Google in the digital contact tracing debate. As a number of public health experts have pointed out, there are good reasons to opt for a centralized approach that have to do with a thorough understanding of contact tracing as a practice involving the norms and skills discussed above, more than with privacy (Kelion 2020, Leprince-Ringuet 2020, Parker et al. 2020).^11^ The most important one goes back to the paucity of the epidemiological data collected by Bluetooth-based contact tracing in comparison to the context-rich data collected by human contact tracers. With a high risk of false positives and false negatives, a centralized approach, according to some public health officials, allows for better supervision and control of these data so that warnings are only sent out to people who have been in epidemiologically significant contact with an infected person. Too many false alarms can quickly result in people not paying attention to warnings sent by an app.

Sphere transgression, here the conversion of technical expertise into an advantage in the sphere of health and medicine, thus risks crowding out practices, norms and values that have been central to this sphere. The deeper the inroads the large tech companies undertake into the health and medical and other sectors, with what might be very efficient, quick and low-cost solutions, the more they also make themselves necessary passage points for the adequate functioning of these sectors, increasing our reliance on them for essential goods. In the context of a pandemic, where human proximity is the primary threat, the dependency on infrastructures for mediated and remote human contact—telehealth, communications services, cloud storage—is amplified (Klein 2020). This can lead to a reshaping of these sectors to align with the values and interests of non-specialist private actors, which may or may not be the interests and values of those groups and individuals who should immediately benefit from the distribution of goods in those spheres, be they patients, students, residents of a city, or more generally speaking, citizens.

## Sphere transgression: from digital goods to public services

### A governance surplus

This conversion of tech expertise into an advantage in the health and medical sphere and other professional sectors also translates into a governance surplus. It grants these actors not just a say in defining the values of a professional sector, but also in the future directions that a sector will take and thus in political decisions concerning society writ large. This is an additional transgression, here into the sphere of politics. Unlike the sometimes aggressive and disruptive practices of tech companies pushing into sectors like health and medicine, this transgression increasingly tends to be a peaceful one, a response to a solicitation on the part of policy makers themselves. In the context of the pandemic, this type of solicitation took place very early on. Already as early as mid-March 2020, representatives from Facebook, Google, Microsoft and Amazon were asked to join several COVID-19 task force meetings at the White House (Robbins 2020). In the UK in the same week, representatives of these companies and Palantir were invited by government officials to present their offers to help tackle the pandemic in terms of data science, app development and data architecture (Volpicelli 2020). And Verily’s first testing sites (and so the creation for its database for COVID-19 related research), were developed in partnership with California state’s governor’s office. In May, Governor Andrew Cuomo of New York, the hardest hit state in the US, announced new partnerships with both Eric Schmidt, former Google CEO, and the Bill and Melinda Gates Foundation (BMGF).^12^ Schmidt will chair a blue-ribbon commission on how to leverage, accelerate and use technology to shape New York’s post-COVID reality, while the BMGF will help develop a blueprint for a “smarter education system”.

Numerous commentators have argued, also in relation to the current pandemic, that at least in the US and the UK, the surge of big tech involvement has much to do with an ongoing privatization, deregulation and reorganization of the public sector (Couldry and Mejias 2018; Klein 2007, 2020; Lawrence et al. 2020; Magalhaes and Couldry 2020; Mazzucato 2015; Morozov 2020). Decades of outsourcing and budget cuts, justified by a dominant political discourse that governments are inefficient, have significantly hampered governments’ abilities to adequately provide health, housing, education, transportation, utilities and other essential services, including, in many countries, pandemic crisis response. This situation has been nourished by a narrative of innovation that has focused on the role of the private sector, and in particular the brilliance of a few individual entrepreneurs, as the drivers of technological development and innovation. As the economist Marianna Mazzucato (2015) maintains, this narrative is both factually incorrect—the state, not the private sector, has traditionally assumed the risks of uncertain technological enterprises that led to the development of amongst others, nanotechnology, biotechnology, the internet, and the iPhone—and it creates a powerful and pernicious self-fulfilling prophecy. In some countries at least, it is thus into a vacuum left over by “rolled back” states that tech corporations easily step in to address problems that governments are currently failing to solve. “We need more Googles,” the governor of California proclaimed after Google announced it would provide 100,000 Wi-Fi hotspots to support remote education in California (in Elias 2020).

### Philanthro-technocapitalism and the need to be grateful

Importantly, in today’s philanthrocapitalism (Bishop and Green 2008), and specifically in today’s philanthro-technocapitalism, it is not at all easy to discern between what are strictly speaking corporate practices and what are giving practices, what are business aims and what are charitable efforts.^13^ These converge in novel ways. Well before being approached by Governor Cuomo, Eric Schmidt had already spelled out his vision for surviving the pandemic in an op-ed piece in the *Wall Street Journal* titled “A Real Digital Infrastructure at Last” (Schmidt 2020). Here, he pleaded for the necessity of accelerating the permanent integration of emerging technologies that are being deployed in the current crisis. Among others, technologies for efficient supply and distribution of goods, for remote education, for health and medicine, and a digital infrastructure to support a future economy and education system “based on tele-everything”. Perhaps most striking was Schmidt’s call on the American population to “be a little more grateful”. He writes: “the benefit of these corporations, which we love to malign, in terms of the ability to communicate, the ability to deal with health, the ability to get information, is profound. Think about what your life would be like without Amazon.”

Schmidt’s plea for more appreciation is not entirely misplaced. Gratitude is to be expected in response to beneficence and charity, and many of the contributions by big tech to the pandemic response have been made as donations or through philanthropic foundations. Gratitude, however, has no place in a social contract. As the sociologist Lindsey McGoey (2015) has poignantly shown in her study of the impact the Bill and Melinda Gates Foundation (BMGF) has had on global health and development, while philanthropies may certainly be well-intentioned and can direct resources to important and often neglected causes, they can have a wide range of negative effects. First, when they are big, they can distort the funding landscape, creating critical energy around one particular area and drawing it away from others.^14^ Second, while the amount of funds donated by a foundation like BMGF to global health may seem significant, it pales in comparison to what governments, sustained by taxes, spend on health—even the poorest ones. Philanthropic donations to vaccine development in the current pandemic, while receiving a lot of media attention, are no exception. It should be clear, furthermore, as McGoey and others point out (Reich 2018; Piketty 2020), that charitable gifts are actually donations which are subsidized, again, by tax payers. Both because philanthropists receive tax privileges for donations, and because the taxes they would have been paying on the part of their wealth that is being donated would have gone to various social programs. For these scholars, understood as subsidized donations, big tech philanthropy sits uncomfortably with the fact that these corporations are partially responsible for generating some of the harm to workers and the environment that they purport to solve through philanthropy. Finally, charity can be withdrawn at the whim of the giver. There is no law that compels wealthy individuals to redistribute any of their wealth in charitable ways. Other than expressions of gratitude, a citizen body has little say in the giving practices of philanthropists; that is precisely the problem. As Saint Augustine professed in earlier times, charity is no substitute for justice withheld (McGoey 2020).

### Contributing to or determining pandemic response?

The decimation of the public sector, and the role this plays in facilitating big tech’s involvement in the political sphere’s pandemic response, is characteristic of the US and the UK more than it is of continental Europe. And indeed, it is mostly in the US and to some extent in the UK that examples of pandemic response partnerships between state or federal actors and tech corporations abound. But it is a mistake to think that the encroachment of these companies into the political sphere is limited to those countries. This, in light of the knock-on effects that the organization of the political sphere in a dominant country like the US has on the rest of the world, and furthermore, in light of the global reach of their operating systems and digital infrastructure (van Dijck et al. 2019; Taylor et al. 2020). In terms of the latter, especially, the Apple/Google API is a case in point.

As explained earlier, the Apple/Google API was presented as a privacy-friendly protocol because it incorporated the main requirements for privacy-preserving contact tracing as expounded by privacy experts; in particular, its adoption of a decentralized model. Its espousal by governments in Europe, furthermore, was in large part influenced by a sustained campaign in favor of decentralized protocols led by activists and academics echoed in statements made by a number of EU bodies. But several reports on discussions that took place between government officials and Apple and Google portray another side of this story, pointing to a power play between sovereign states and the corporations, in which sovereign states had little say. Namely, some government officials claim to have been taken by surprise by the development of the ready-made API at a time when they were already far advanced with their own protocols. Others have depicted a situation in which there was little possibility to shape how the protocol would be adapted to their respective countries.

In France, for example, which had been working on a centralized protocol, officials have reported that when they found out about the Apple/Google API and tried to approach the companies to find workarounds, their attempts were met with staunch reaffirmations that the companies would only work with decentralized technologies (Scott et al. 2020). For a country like France, which insisted on pursuing its national centralized system, this meant open confrontation with the tech companies, and being portrayed in the media as caring less about privacy than the tech companies did (Hern 2020). Similarly, a representative of the Latvian government, which has adopted the Apple/Google API, has openly described discussions with the companies as running “into a Silicon Valley-built brick wall” and has questioned the extent to which Google or Apple should “get to tell a democratically elected government or its public health institutions what they may or may not have on an app” (Ilves 2020). Such frustrations around the need to comply with the rules set out by the companies have been echoed in other countries and federal authorities as well, including the UK and North Dakota in the US (Tometzkis and Meaker 2020). Namely the interoperability gains that come with collaborating with the companies that run the two most important mobile operating systems on the planet meant that going it alone could compromise success. The Apple/Google API is thus also an example of encroachment into the political sphere—here global public health policy. Effectively, Apple and Google did not just contribute their technical expertise to the pandemic response, but also determined—in some instances over and above sovereign states—which path to take, setting down the conditions for which apps could exist and how governments could use them.

## Conclusion

This article has attempted to articulate what is at stake when two of the world’s most powerful corporations move into the field of pandemic response management with a development like the Apple/Google API for digital contact tracing. I argued that this is an instance of illegitimate sector creep, or sphere transgression. In this case, a legitimate advantage acquired in the sphere of digital goods—digital expertise—has been converted into advantages in the sphere of health and medicine (where epidemiological expertise should be the main source of legitimacy), and in the sphere of politics (where democratic accountability should be the source of legitimacy). Each of these transgressions presents its own risks. Namely, a crowding out of essential spherical expertise, new dependencies on corporate actors for the delivery of essential, public goods, the shaping of (global) public policy by non-representative, private actors and ultimately, the accumulation of decision-making power across multiple spheres. Such sphere transgressions are not novel to the Apple/Google API case, nor are they limited to these two companies. Rather, they can be identified as a defining characteristic of the digitalization and datafication of society that has been underway in recent decades. Indeed, as more and more sectors of society undergo processes of digitalization, digital expertise becomes an entry ticket to previously autonomous spheres, bringing with it other values and interests and granting newfound power to reshape spheres according to those values and interests.

Sphere transgression can happen in perfectly privacy-friendly ways, such that, as argued, the focus on how privacy-preserving the Apple/Google API is misses the point. Moreover, as these companies increasingly incorporate privacy considerations in their tech development, we need to ask ourselves how privacy-friendliness may actually *facilitate* sphere transgression, lest our sharpest critical engagements end up weakening rather than strengthening our democracies. This is not to say that the push for privacy in contact tracing and other digital practices is not important, but that when actors like Apple and Google are involved we need a much broader lens through which to consider benefits and trade-offs, and with which to develop new safeguards. Complete sphere autonomy may not be a desirable or feasible solution for a world so deeply connected, and where digital infrastructures and expertise have become so essential. Spheres clearly overlap at times, certainly during a pandemic: to be sure, digital contact tracing can be an important complement within a broad pandemic response strategy, and to be done properly it requires a combination of both epidemiological and technical expertise. Nevertheless, new ways of managing migrations between spheres can and should be developed. By setting down pre-conditions for sphere penetration, in law, professional practice and oversight, we may find the power to protect traditional spherical expertise, enable beneficial combinations of expertise, and keep power in check.

## Footnotes

1 For early June 2020, according to *The Correspondent’s* “Track(ed) Together” database. See https://thecorrespondent.com/collection/track-ed-together.

2 Privacy is not the only ethical concern that has been voiced by legal scholars, ethicists and activists. Others include fair data sharing practices, responsible data use, discrimination, freedom of movement and voluntariness. See for example Lucivero et al. (2020) and Morley et al. (2020). Furthermore, this article focusses on the contact tracing debate and the involvement of large tech companies in pandemic response measures in the context of the US and Europe. For an overview of COVID-19 responses in the global setting and in relation to global justice, see the volume edited by Taylor et al. (2020).

3 China’s AliPay Health Code app, one of the first contact tracing apps, is being used in this way. In the Netherlands, a QR based app is being used to allow access to some restaurant terraces (https://checkgesprek.nl).

4 See for example, the joint statement signed by over 300 academics internationally in support of decentralized protocols: https://giuper.github.io/JointStatement.pdf. Much of this discussion played out on Twitter: https://twitter.com/mikarv/status/1252213057213915136?s=20

5 https://twitter.com/EU_EDPS/status/1248661369274150912.

6 See MIT Technology Review’s ‘Covid Tracking Tracker’. https://www.technologyreview.com/2020/05/07/1000961/launching-mittr-covid-tracing-tracker/

7 Some critics of Bluetooth-based contact tracing are instead advocating for QR-code based contact tracing, which requires much more intentionality on the part of users, who have to scan QR codes to enter certain spaces. See for example Hoffman et al.’s piece on the “Zwaai” QR-code based app in this issue.

8 Gates is often quoted as having anticipated that a pandemic similar to coronavirus would sweep the globe in a TED talk in 2015.

9 For a more detailed explanation of how this framework can be applied in this context, and why this amounts to more than just the encroachment of the market sphere into the sphere of health and medicine, see Sharon (2020). There I show how a multiple sphere ontology and the notion of sphere transgression based on illegitimate conversions of advantages of one into other spheres sheds light on additional risks raised by the Googlization of health. These have to do not only with an importation of a marketplace logic into the sphere of health and medicine – although this is the most evident risk – but also with the importation of an “industrial” logic of efficiency, a “civic” logic of democratization of knowledge, a “project” logic of innovation for innovation’s sake, and possibly others.

10 Though developing inhouse medical expertise, by hiring leading medical specialists into their ranks, is a strategy that is being pursued by these companies alongside the new partnerships they are establishing with traditional institutions and sectorial actors (Check 2015; Reisinger 2018). For an analysis of how these new partnerships are being justified, see Sharon (2018).

11 While some proponents of centralized tracing also argue that there is no reason per se why privacy and data security cannot be preserved in a centralized approach (Ilves 2020). Just as data collected by health authorities via manual tracing need not reveal the identity of infected persons, explore the nature of that contact or be shared with third parties, neither must this necessarily be the case in digital contact tracing.

12 See https://twitter.com/CNNnewsroom/status/1258065906061791233 and https://www.governor.ny.gov/news/amid-ongoing-covid-19-pandemic-governor-cuomo-announces-collaboration-gates-foundation-develop.

13 Apple CEO Tim Cook has been very explicit about how commercial and altruistic objectives converge at Apple. When recently asked in an interview why Apple has not set up a charitable foundation like other companies, his answer was that setting up a foundation separates a company’s capacity to produce social value from its core business. This, as he perceives it to be the case with Apple, should be integral to, not disconnected from what a company does (Cook 2019).

14 McGoey cites the example of charter schools in the US, which the BMGF supported, and which have contributed, according to some analysts, to an increase in educational inequalities.

## References

[CR1] Ada Lovelace Institute. (2020). *Exit through the app store*. https://www.adalovelaceinstitute.org/our-work/covid-19/covid-19-exit-through-the-app-store/. Accessed 25 June 2020.

[CR2] Amazon. (2020). Amazon donating $5 million in devices globally. April 10. https://blog.aboutamazon.com/devices/amazon-donating-5-million-in-devices-globally. Accessed 25 June 2020.

[CR3] Apple. (2020). *Privacy-preserving contact tracing*. https://www.apple.com/covid19/contacttracing. Accessed 25 June 2020.

[CR4] Ball K, Snider L (2013). The surveillance-industrial complex: A political economy of surveillance.

[CR5] Berg M (1997). Rationalizing medical work: Decision support techniques and medical practices.

[CR6] Bishop M, Green M (2008). Philanthrocapitalism: How the rich can save the world.

[CR8] Centers for Disease Control and Prevention (CDC). (2020a). *Preliminary criteria for the evaluation of digital contact tracing tools for COVID-19.* https://www.cdc.gov/coronavirus/2019-ncov/downloads/php/prelim-eval-criteria-digital-contact-tracing.pdf. Accessed 25 June 2020.

[CR9] Centers for Disease Control and Prevention (CDC). (2020b). Digital contact tracing tools for COVID-19. https://www.cdc.gov/coronavirus/2019-ncov/downloads/digital-contact-tracing.pdf. Accessed 25 June 2020.

[CR10] Cerulus, L. (2020). Huwaei joins China’s Big Tech donation spree in Europe. *Politico*, March 25. https://www.politico.eu/article/huawei-joins-chinas-big-tech-donation-spree-in-europe/. Accessed 25 June 2020.

[CR11] Check H (2015). Why biomedical superstars are signing on with Google. Nature.

[CR12] European Commission (EC) (2020). *Commission Recommendation (EU) 2020/518 of 8 April 2020.* Available at https://ec.europa.eu/commission/presscorner/detail/en/ip_20_626. Accessed 25 June 2020.

[CR13] Cook, T. (2019*). *Interview with J. Cramer. *CNBC* https://www.cnbc.com/2019/01/08/apple-ceo-tim-cook-interview-cnbc-jim-cramer-transcript.html?__source=twitter%7Cmain. Accessed 25 June 2020.

[CR14] Couldry N, Mejias U (2018). Data colonialism: Rethinking big data’s relation to the contemporary subject. Television & New Media.

[CR15] Nuffield Council on Bioethics (NCB). (2020). *Ethical Considerations in Responding to the COVID-19 Pandemic.* https://www.nuffieldbioethics.org/assets/pdfs/Ethical-considerations-in-responding-to-the-COVID-19-pandemic.pdf

[CR16] Counterpoint Research. (2020). *Coronavirus: Why are there doubts over contact-tracing apps?* https://www.counterpointresearch.com/coronavirus-doubts-contact-tracing-apps/. Accessed 25 June 2020.

[CR17] Criddle, C. and Kelion, L. (2020). Corona virus contact-tracing: The world split between two types of apps. *BBC,* May 7. https://www.bbc.com/news/technology-52355028. Accessed 25 June 2020.

[CR18] Dave, P. (2020). WHO readies coronavirus app for checking symptoms, possibly contact tracing. Reuters, May 9. https://uk.reuters.com/article/health-coronavirus-who-apps/who-readies-coronavirus-app-for-checking-symptoms-possibly-contact-tracing-idUKKBN22L06L. Accessed 25 June 2020.

[CR19] DeepMind. (2020). *Computational predictions of protein structures associated with COVID-19*. April 8. https://deepmind.com/research/open-source/computational-predictions-of-protein-structures-associated-with-COVID-19?ref=hackernoon.com. Accessed 25 June 2020.

[CR20] The Economist. (2020). Countries are using apps and data networks to keep tabs on the pandemic. *The Economist,* March 26. https://www.economist.com/briefing/2020/03/26/countries-are-using-apps-and-data-networks-to-keep-tabs-on-the-pandemic. Accessed 25 June 2020.

[CR21] EDPS (European Data Protection Supervisor). (2020). *EU digital solidarity: A call for a pan-European approach against the pandemic*. https://edps.europa.eu/sites/edp/files/publication/2020-04-06_eu_digital_solidarity_covid19_en.pdf. Accessed 25 June 2020.

[CR22] Elias, J. (2020). California governor says, ‘We need more Googles’ as company offers free Wi-Fi and Chromebooks to students. *CNBC* April 1. https://www.cnbc.com/2020/04/01/coronavirus-google-offers-wi-fi-chromebooks-to-california-students.html. Accessed 25 June 2020.

[CR23] Facebook. (2020). *Data for Good.* https://dataforgood.fb.com. Accessed 25 June 2020.

[CR24] Ferreti L (2020). Quantifying SARS-CoV-2 transmission suggests epidemic control with digital contact tracing. Science.

[CR25] Fitzgerald, M. and Crider, C. (2020). Under pressure, UK government releases NHS COVID data deals with big tech. *OpenDemocracy* June 5. https://www.opendemocracy.net/en/under-pressure-uk-government-releases-nhs-covid-data-deals-big-tech/. Accessed 25 June 2020.

[CR26] Frieden, T. (2020). A video conversation on the coronavirus with Tom Frieden with *STAT’s* Helen Branswell. https://www.statnews.com/2020/04/13/video-chat-conversation-on-the-coronavirus-with-tom-frieden/. Accessed 25 June 2020.

[CR27] Frohlich H (2018). From hype to reality: Data science enabling persnalized medicine. BMC Medicine.

[CR29] Goldenfein, J., Green, B., Viljoen, S. (2020). Privacy versus health is a false trade-off. *Jacobin* April 17. https://jacobinmag.com/2020/04/privacy-health-surveillance-coronavirus-pandemic-technology. Accessed 25 June 2020.

[CR30] Grothaus, M. (2020). Bill Gates announces his foundation will focus ‘total attention’ on COID-19 pandemic. *Fast Company*, April 27. https://www.fastcompany.com/90497398/bill-gates-announces-his-foundation-will-focus-total-attention-on-covid-19-pandemic. Accessed 25 June 2020.

[CR31] Hern, A. (2020). France urges Apple and Google to ease privacy rules on contact tracing. *The Guardian*, April 21. https://www.theguardian.com/world/2020/apr/21/france-apple-google-privacy-contact-tracing-coronavirus. Accessed 25 June 2020.

[CR32] Hildebrandt M, Tielemans L (2013). Data protection by design and technology neutral law. Computer Law and Security Review.

[CR33] Hinch, R. et al. (2020). *Effective configurations of a digital contact tracing app: A report to NHSX*, 14 April 2020. https://github.com/BDI-pathogens/covid-19_instant_tracing/blob/master/Report%2520-%2520Effective%2520Configurations%2520of%2520a%2520Digital%2520Contact%2520Tracing%2520App.pdf. Accessed 25 June 2020.

[CR34] Hoepman, J.H. (2020). Stop the Apple and Google contact tracing platform (or be ready to ditch your smartphone). https://blog.xot.nl/2020/04/11/stop-the-apple-and-google-contact-tracing-platform-or-be-ready-to-ditch-your-smartphone/. Accessed 25 June 2020.

[CR35] Ienca M, Vayena E (2020). On the responsible use of digital data to tackle the COVID-19 pandemic. Nature Medicine.

[CR36] Ilves, I. (2020). Why are Google and Apple dictating how European democracies fight coronavirus? *The Guardian*, June 16. https://www.theguardian.com/commentisfree/2020/jun/16/google-apple-dictating-european-democracies-coronavirus?CMP=Share_AndroidApp_Tweet. Accessed 25 June 2020.

[CR37] Kelion, L. (2020). NHS rejects Apple-Google coronavirus app plan. *BBC*, April 27. https://www.bbc.com/news/technology-52441428. Accessed 25 June 2020.

[CR38] Klein M (2007). The shock doctrine: The rise of disaster capitalism.

[CR39] Klein, M. (2020). Screen New Deal. *The Intercept*, May 8. https://theintercept.com/2020/05/08/andrew-cuomo-eric-schmidt-coronavirus-tech-shock-doctrine/. Accessed 25 June 2020.

[CR40] Kurian, T. (2020). How Google Cloud is helping during COID-19. https://cloud.google.com/blog/topics/inside-google-cloud/how-google-cloud-is-helping-during-covid-19. Accessed 25 June 2020.

[CR41] Lawrence, F. et al. (2020). How a decade of privatization and cuts exposed England to coronavirus. *The Guardian,* May 31. https://www.theguardian.com/world/2020/may/31/how-a-decade-of-privatisation-and-cuts-exposed-england-to-coronavirus. Accessed 25 June 2020.

[CR42] Lee, A. (2020). If Bluetooth doesn’t work for contact-tracing apps, what will? *Wired*, April 17. https://www.wired.co.uk/article/bluetooth-contact-tracing-apps. Accessed 25 June 2020.

[CR43] Leprince-Ringuet, D. (2020). The world’s first contact-tracing app using Google and Apple’s API goes live. *ZDNet*, May 28. https://www.zdnet.com/article/the-worlds-first-contact-tracing-app-using-google-and-apples-api-goes-live/. Accessed 25 June 2020.

[CR44] Leswing, K. (2020). Stanford teamed up with Apple to release an app that connects first responders to drive-through COVID-19 tests. *CNBC*, April 9. https://www.cnbc.com/2020/04/09/stanford-apple-app-connects-first-responders-to-covid-19-tests.html. Accessed 25 June 2020.

[CR45] Lucivero, F., Hallowell, N., and Johnson, S., Prainsack, B., Samuel, G., and Sharon, T. (2020). Covid-19 and Contact Tracing Apps: Ethical challenges for a social experiment on a global scale. *Journal of Bioethical Inquiry.* 10.1007/s11673-020-10016-9 PMC7445718 32840842

[CR46] Magalhaes, J.C. and Couldry, N. (2020). Tech giants are using this crisis to colonize the welfare system. *Jacobin*, April 27. https://www.jacobinmag.com/2020/04/tech-giants-coronavirus-pandemic-welfare-surveillance. Accessed 25 June 2020.

[CR47] Mazzucato M (2015). The entrepreneurial state: Debunking public vs. private sector myths.

[CR48] McGee, P., Murphy, H. and Bradshaw, T. (2020). Coronavirus apps: the risk of slipping in to a surveillance state. *Financial Times*, April 28 https://www.ft.com/content/d2609e26-8875-11ea-a01c-a28a3e3fbd33. Accessed 25 June 2020.

[CR49] McGoey L (2015). No such thing as a free gift: The gates foundation and the price of philanthropy.

[CR50] McGoey, L. (2020). Bill Gates and the price of philanthropy: An interview with activism Munich. https://www.youtube.com/watch?v=ruR5FK8HN5Q

[CR51] Meaker, M. and Tokmetzis, D. (2020). Coronavirus apps show governments can no longer do without Apple or Google. *The Correspondent*, June 22. https://thecorrespondent.com/546/coronavirus-apps-show-governments-can-no-longer-do-without-apple-or-google/417112964268-93dd1b76. Accessed 25 June 2020.

[CR52] Mol A (2008). The logic of care: Health and the problem of patient choice.

[CR53] Morley J, Cowls J, Taddeo M, Floridi L (2020). Ethical guidelines for COVID-19 tracing apps. Nature.

[CR54] Morozov, E. (2020). The tech ‘solutions’ for coronavirus take the surveillance state to the next level. *The Guardian*, April 15. https://www.theguardian.com/commentisfree/2020/apr/15/tech-coronavirus-surveilance-state-digital-disrupt. Accessed 25 June 2020.

[CR55] Nissenbaum H (2010). Privacy in context.

[CR56] O’Neil, C. (2020). The Covid-19 tracking app won’t work. *Bloomberg* 16 April. https://www.bloomberg.com/opinion/articles/2020-04-15/the-covid-19-tracking-app-won-t-work. Accessed 25 June 2020.

[CR57] Otterman, S. (2020). As the Nation Begins Virus Tracing, it Could Learn from this N.J. City. *The New York Times*, May 21. https://www.nytimes.com/2020/05/21/nyregion/contact-tracing-paterson-nj.html. Accessed 25 June 2020.

[CR58] Parker, I. and Jones, E. (2020). Something to declare? Surfacing issues with immunity certificates. https://www.adalovelaceinstitute.org/something-to-declare-surfacing-issues-with-immunity-certificates/. Accessed 25 June 2020.

[CR59] Parker M (2020). Ethics of instantaneous contact tracing using mobile phone apps in the control of the COVID-19 pandemic. Journal of Medical Ethics.

[CR60] European Parliament. (2020). EU coordinated action to combat the COVID-19 pandemic and its consequences. https://www.europarl.europa.eu/doceo/document/TA-9-2020-0054_EN.pdf. Accessed 25 June 2020.

[CR61] Piketty T (2020). Capital and ideology.

[CR62] Piper, K. (2020). Bill Gates’s efforts to fight coronavirus, explained. *Vox*, May 4. https://www.vox.com/future-perfect/2020/4/14/21215592/bill-gates-coronavirus-vaccines-treatments-billionaires. Accessed 25 June 2020.

[CR63] Pols J (2012). Care at a distance: On the closeness of technology.

[CR64] Rahman, Z. (2020). Black Lives Matter protester aren’t being tracked with Covid-19 surveillance tech. Not yet. *The Correspondent*, June 3. https://thecorrespondent.com/507/black-lives-matter-protesters-arent-being-tracked-with-covid-19-surveillance-tech-not-yet/569187644025-767f5154. Accessed 25 June 2020.

[CR65] Reich R (2018). Why philanthropy is failing democracy and how it can do better.

[CR66] Reisinger, D. (2018). Apple has hired nearly 50 medical doctors in wellness push. *Fortune*, December 13. https://fortune.com/2018/12/13/apple-hires-medical-doctors/. Accessed 25 June 2020.

[CR67] Robbins, R. (2020). The White House is pinning its hopes on health tech to save the day. Can it deliver? *STAT*, March 18. https://www.statnews.com/2020/03/18/coronavirus-white-house-pinning-hopes-on-health-tech/?utm_source=STAT+Newsletters&utm_campaign=e4b0e3649e-health_tech_COPY_01&utm_medium=email&utm_term=0_8cab1d7961-e4b0e3649e-151653869. Accessed 25 June 2020.

[CR68] Ross, C. (2020). 5 burning questions about tech efforts to track Covid-19 cases. *STAT* April 18. https://www.statnews.com/2020/04/15/coronavirus-digital-contact-tracing-tech-questions/. Accessed 25 June 2020.

[CR69] Roth, A. et al. (2020). Growth in surveillance may be hard to scale back after pandemic, experts say. *The Guardian*, April 14. https://www.theguardian.com/world/2020/apr/14/growth-in-surveillance-may-be-hard-to-scale-back-after-coronavirus-pandemic-experts-say. Accessed 25 June 2020.

[CR70] Schmidt, E. (2020). A real digital infrastructure at last. *Wall Street Journal*, March 27. https://www.wsj.com/articles/a-real-digital-infrastructure-at-last-11585313825. Accessed 25 June 2020.

[CR71] Schwartz, A. (2020). How EFF evaluates government demands for new surveillance. https://www.eff.org/deeplinks/2020/04/how-eff-evaluates-government-demands-new-surveillance-powers. Accessed 25 June 2020.

[CR72] Scott, M, Braun, E. Delcker, J. and Manancourt, V. (2020). How Google and Apple outflanked governments in the race to build coronavirus apps. *Politico*, May 15. https://www.politico.eu/article/google-apple-coronavirus-app-privacy-uk-france-germany/. Accessed 25 June 2020.

[CR74] Sharon T (2016). The Googlization of health research: From disruptive innovation to disruptive ethics. Personalized Medicine.

[CR75] Sharon T (2018). When digital health meets digital capitalism, how many common goods are at stake?. Big Data & Society.

[CR76] Sharon, T. (2020). Beyond hostile worlds: The multiple sphere ontology of the digitalization and Googlization of health. https://papers.ssrn.com/sol3/papers.cfm?abstract_id=3633371

[CR77] Taylor L, Sharma G, Martin A, Jameson S (2020). Global data justice and COVID-19.

[CR78] Timmermans S, Mauck A (2005). The promises and pitfalls of evidence-based medicine. Health Affairs.

[CR79] Tometzkis, D. and Meaker, M. (2020). We were told technology would end Covid-19 lockdowns, but the truth is there’s no app for that. *The Correspondent*. https://thecorrespondent.com/502/we-were-told-technology-would-end-covid-19-lockdowns-but-the-truth-is-theres-no-app-for-that/66389901600-2c9929bb. Accessed 3 June 2020.

[CR80] Troncoso, C. et al. (2020). White paper on Decentralized Privacy-Preserving Proximity Tracing. https://github.com/DP-3T/documents/blob/master/DP3T%2520White%2520Paper.pdf. Accessed 25 June 2020.

[CR87] Vaidhyanathan S (2011). The Googlization of Everything (and Why We Should Worry).

[CR86] van Dijck J, Poell T, de Waal M (2019). The Platform Society: Public Values in a Connected World.

[CR81] Vaughn, A. (2020). Bluetooth may not work well enough to trace coronavirus contacts. *New Scientist*, May 12. https://www.newscientist.com/article/2243137-bluetooth-may-not-work-well-enough-to-trace-coronavirus-contacts/. Accessed 25 June 2020.

[CR82] Verily. (2020). New Baseline COVID-19 Research Project launches to advance scientific understanding of virus, with initial focus on antibody testing. May 18 https://blog.verily.com/2020/05/new-baseline-covid-19-research-project.html?utm_source=STAT+Newsletters&utm_campaign=3fdf688da3-health_tech_COPY_01&utm_medium=email&utm_term=0_8cab1d7961-3fdf688da3-151653869. Accessed 25 June 2020.

[CR83] Volpicelli, G. (2020). Inside Dominic Cumming’s coronavirus meeting with big tech. *WIRED*, March 12. https://www.wired.co.uk/article/dominic-cummings-coronavirus-big-tech. Accessed 25 June 2020.

[CR84] Walzer M (1983). Spheres of justice: A defense of pluralism and equality.

[CR85] World Health Organization (WHO). (2020). *Digital tools for COVID-19 contact tracing*. https://www.who.int/publications/i/item/WHO-2019-nCoV-Contact_Tracing-Tools_Annex-2020.1. Accessed 25 June 2020.

